# Older Adults Looking for a Job through Employment Support System in Tokyo

**DOI:** 10.1371/journal.pone.0159713

**Published:** 2016-07-21

**Authors:** Ushio Minami, Hiroyuki Suzuki, Masataka Kuraoka, Takashi Koike, Erika Kobayashi, Yoshinori Fujiwara

**Affiliations:** Research Team for Social Participation and Community Health, Tokyo Metropolitan Institute of Gerontology, Tokyo, Japan; Iowa State University, UNITED STATES

## Abstract

**Purpose of the Study:**

This study aims to clarify the job seeking process of the elderly people through the local employment support facility known as the Active Senior Employment Support Center (ASESC)”AKUTIBU SINIA SHUGYO SIEN SENTAA” in the Tokyo metropolitan area, and evaluate the performance as a complement to the national support systems.

**Methods:**

We conducted 6 waves of longitudinal mail surveys over 38 weeks to 235 older job seekers (146 males and 89 females, average age 63.7, SD 5.6), who visited two ASESCs for the first time, to clarify their living situation, health condition, and changes in their job seeking process.

**Results:**

These older job seekers tended to be at a relatively low education level and on low income, as well as tended to seek jobs for earning living expenses rather than for well-being. Half of them found employment in 35.0 days; however, 23.8% couldn’t find any job in 38 weeks, especially those who were younger and with higher education.

**Implications:**

ASESCs are functioning to assist older job seekers who are mainly seeking jobs for earning living expenses, which can be attained in a short time span and enable them to earn some money. These facilities are expected to be consulting services, not only for employment support but also for general living, because it is important to maintain contact with people who are at risk of social isolation, serious financial difficulty, or suicide. We consider it very helpful to encourage and re-activate these mismatched people, by supporting them to engage in highly contributional services to our society and the next generation, such as providing child-care support or daily life support, the demands for which are rapidly increasing due to recent governmental policies.

## Introduction

Due to the progress of a super-aging society, social security expenses are expanding rapidly, whereas general labor forces are decreasing in Japan [1][2]. In addition, the proportion of non-regular employees has increased, and there has been a trend toward wider income disparities [3]. The number of people who will not receive enough pension in their old age is regarded to be increasing. Working in old age is expected not only for economic growth [4] or well-being, but also as a preventive measure against poverty and social isolation.

According to the Statistics Bureau [5], the rate of employment in elderly people aged 65 years and over in Japan (20.8%) is the highest among the Group of Eight industrialized nations. A previous study suggested that the reason for this is because there is a tradition both to respect working people and to regard working as a way of maintaining well-being in Japan, which is different from western countries where people tend to regard working as a kind of punishment [6]. After the 1950s, employees in Japanese society used to have a lifelong commitment to their first employment, until mandatory retirement, probably because the custom of changing jobs was not common in Japan. Unemployment and the job-searching processes of elderly individuals haven’t been academically well examined. A lot of research has been conducted on the effects of retirement [7], but not yet about the process of job searching in old age.

The “Revision of the Act on Stabilization of Employment of Elderly Persons in 2012” has been enforced from April 2013; therefore, those who hope to work until 65 years of age are protected to do so [8]. However, this law is only for regular workers, not for non-regular workers, or unemployed people of involuntary retirement due to either bankruptcy of their former workplace or the responsibility to take care of a family member. In Japan, we have experienced rapid ups and downs of the economy over the last 20 years. This has drastically changed the employment environment, generating either many willing or unwilling early retirees, or other flexible and diversified types of employment such as non-regular workers [9]. It can be said that the employment environment has changed drastically compared with before. Therefore, nowadays, non-regular workers may have to seek new jobs in old age. Especially in Tokyo, the capital of Japan, it is expected that the influence of this change will become rapidly apparent. Tokyo accounts for approximately one-tenth of the population of Japan. The aging rate (proportion of people over 65 years old) in Tokyo (22.8%) is lower than the national average (26.0%) at present [1]; however, the aging rate will accelerate rapidly hereafter. There is a concern that the areas where many elderly people gather may become ghettos [10]. At present, there are many older people who are living in rented houses in Tokyo. As the population is decreasing, the growth rate will decline, and the saving rate and balance of pensions will worsen, meaning many older adults may be unable to afford to pay their rent, and may lose their houses. Moreover, as there is an apparent shortage of nursing facilities for the elderly with dementia in Tokyo recently, older people may become like refugees in their communities. Furthermore, public infrastructure or services may not be maintained or well operated, because of the poor financial situation of local public bodies, and the public safety will get worse.

According to the national census [11], there were 630,613 working people aged 65 and over in Tokyo, which is 23.9% of the population. Of them, 207,400 (32.9%) were self-employed and 120,274 (19.0%) were company executives. The other approximately 303,000 (48.1%) were regarded as regular, dispatched, contract, or part-time workers. However, the process of job-seeking is not yet clear, as well as what kind of people can find what type of job in Japan.

### Employment Support Services for Older Job Seekers in Japan

Currently, there are 3 kinds of services for older job seekers in Tokyo. The most common facility with a wide area network is “Hello-Work”, which is a nationwide public employment service with no age restrictions. There are 550 locations, and the number of new job applicants reached 6.7 million yearly throughout Japan. The number of successful placements for people over 60 years old was about 207,000 in 2013. However, this is only 6.5% of the people who visited Hello-Work [3]. It is prohibited for companies to state age restrictions on job offering forms at Hello-Work according to the amendment of Article 10 in Employment Measures Act from October 2007 [12]. Legally, a company must consider candidates based on their background, skills or experience, not age. In reality, some companies are looking for employees of a certain age for a certain position, leading to the situation in which elderly applicants must write their resumes many times for positions for which they will not even be considered. Though many older job seekers visit Hello-Work first for job-seeking information, it often doesn’t help them, and many of them give up trying to find a job through Hello-Work.

The second network is the Silver Human Resource Center (SHRC). There are 1299 locations and 743,969 older people are registered [13]. This network is the most popular and common way for older people in Japan who hope to work for their well-being [14] and not for earning living expenses. This will provide opportunities to participate not only in jobs but also in many kinds of hobby circles, volunteer activities, or vocational trainings for older people. However, the number of employments referred by SHRC was only about 65,965 in Tokyo, which is about 10.5% of the working people aged 65 years and over. The jobs the members engage in are restricted to low wage and light work, up to 20 hours a week. The average wage is about 316,427 Japanese yen yearly [15], which is about one fifth of the welfare support for public assistance recipients, defined as the expense for maintaining the minimum standard of wholesome and cultured living by the Constitution of Japan. The average age of the registered members is gradually becoming higher, to over 70 years old, and the number of members has been decreasing in recent years. Older job seekers who hope to work for their well-being, would be able to get a job through SHRC, but it is not suitable for younger elderly people who are healthy enough to work, and want to earn more.

The third alternative is the Active Senior Employment Support Center (ASESC)”AKUTIIBU SINIA SHUGYO SIEN SENTAA” which is for job seekers aged 55 and over. This center is located in 10 places in the Tokyo metropolitan area. It introduces local job-offers near each facility and there is no limit to the total weekly working hours. ASESCs are usually located in small spaces, and managed by a small staff. ASESCs were previously called “free employment agencies”, which were established by the Tokyo local government in 1963 and have continued operating while changing the target age, area, and administration over time [16].

This study aims to clarify the performance of this third facility, ASESC, which is managed by the Social Welfare Council and sponsored by the Tokyo Metropolitan Government and ward offices. The contribution of these facilities has not yet been well studied. ASESC has mobility and flexibility on a small scale and is expected to complement the previously-mentioned national networks, Hello-Work and SHRC, in order to discover the potential demand in the local community for older workers, which the other networks have not yet tapped. Some examples of demanding jobs for older adults include working as contingent staff on bargain days at a shopping street, cleaning staff or a guard at a community park event, a house-to-house visitor collecting survey questionnaires, an attendant helping children commute to kindergarten, or support staff working in a small factory in town during a busy season. In this field, there may be a better opportunity for people who are more diligent, more reliable, or better-known among local people in a community, compared with younger people, and are better able to sympathize with the situation of others. Also, there may be a chance for people who can work in a flexible working style, even for a short time, with specialized skills; those who are not reluctant to try a small start-up or an unstable business (who can take a risk); or those who can pay more attention to contributing to our society or public interests.

In this study, the employment support for older job seekers was considered in ASESC, from the view point of welfare for elderly people [7], in order to remark on the superiority of older adults and to sustain their dignity or well-being, and not for merely treating them as low-wage manual workers.

## Materials and Methods

### Data Collection

Longitudinal mail questionnaire surveys were conducted 6 times on the older job seekers who visited 2 ASESCs of the same scale of floor space, number of staff, and territory, both being located in the Tokyo metropolitan area. One is on the north side of Tokyo, while the other is on the south side. According to statistics open to the public, which were not related to this study sample, but gave rough estimates about the number of job offers and job seekers dealt with at each facility, the cumulative total number of visitors to one of the two facilities was 2,849, which included people who didn’t register as a job seeker and multiple visits by one person. Of the 652 job offers, 202 older job seekers could find a job in 2012 [17]. At the other facility, there were 2,863 cumulative visitors, where of the 575 job offers, 180 persons were employed in 2011 [18].

We asked the ASESC staff at these 2 facilities to hand out the first questionnaire (Baseline Survey: BL) randomly to the visitors who were registered as job seekers for the first time, to clarify their living situation or health condition, over the span of January 28, 2013 to March 31, 2015. Moreover, we conducted five follow-up surveys (F1-F5) after 2, 6, 14, 26, 38 weeks by mail to each person separately who responded to the first questionnaire to clarify changes in their job-seeking status. The number of questionnaires handed out at the two centers was 387. Among them, the number of respondents to the BL was 235 people (Response Rate: 60.7%). The number of respondents to the following investigations are as follows; F1:197 (83.8%), F2:179 (90.9%), F3:158 (88.3%), F4:144 (91.1%), F5:130 (90.3%).

This study sample doesn’t represent all older job seekers in Tokyo, but only the visitors at ASESC which we regard, as an effective complement to the former-mentioned national services, Hello-Work and SHRC.

### Ethical Considerations

This research has been approved by the Ethics Committee (IRB: Institutional Review Board) of the Tokyo Metropolitan Institute of Gerontology. Participants were explicitly informed that the data produced in the survey would be confidential, would not affect the provision of employment support services to them, and would be used only for academic research purposes. We regarded the return of the questionnaire as the informed consent of the respondents, as instructed at its beginning. We provided a reward of a voucher on reply in the longitudinal study.

### Measures

The following variables were examined to measure the situation of each older job seeker; 4 variables of basic attributes: sex, age, education, and household income; 3 variables of life circumstance: social isolation (which was defined as when the total number of meetings, outings, calling or mailings with family members, relatives, friends, or neighbors were less than one time per week [19]), social participation (as a member of a neighborhood social group, volunteer, hobby, etc.), and living alone; 2 variables of health status: Self-rated health and WHO5 well-being index [20]; and 3 variables about job-seeking: reason for job-seeking within 8 alternatives (multiple answers), hoped-for occupations beforehand within 12 alternatives (multiple answers), and status of working (working or not working, single answer). All variables were categorized for statistical analysis.

### Analysis

In the analysis, we showed the characteristics of respondents, at first, using the responses of 235 people (146 males and 89 females, average age 63.7, SD 5.6) as a baseline survey by chi-square tests. Also, to examine the performance of attainment (rate of employment), we targeted the 172 people who didn’t have a job during the baseline survey (73.5% of BL, 109 males and 63 females, average age 63.3, SD5.4) and used the Kaplan-Meier Estimator with Log-Rank tests, setting the statuses of working, from each survey, as objective variables. During the research period, 131 people found employment. Once they were employed, we stopped the follow-up and excluded them from analysis even though the survey continued. By doing so, we estimated the period to find a new job by older job seekers who first visited the facility and didn’t have a job. It was also confirmed by the Cox proportional-hazard regression model for job matching by the job seekers’ attributes.

The Kaplan-Meier Estimator with Log-Rank tests and the Cox proportional-hazard regression model are survival analyses for this kind of incomplete data including censoring, missing, or nondisclosure [21]. These methods are often used in medical statistics to examine the time-series effects after treatment represented as a binary code or exclusive categories (e.g., the dosage of medicine). These methods have been used widely to analyze the state of job loss and attainment [22] using survival curve in reverse.

Lastly, we compared the hoped-for occupations beforehand and the occupation actually hired as. We used SPSS version 20 for all analyses.

## Results

Table 1 shows the baseline characteristics of the respondents. There were significantly more men who were socially isolated and with poor mental health measured by WHO5, compared with women. The most common reason for job seeking in these ASESCs was for earning living expenses, followed by maintaining health, well-being, and social participation. In particular, there were more women seeking jobs for well-being or social participation than men. The mean period of job seeking before arriving at ASESC was 5.75 months, and 65.5% of those surveyed had previously used the services of Hello-Work.

**Table 1:** Baseline Characteristics of Older Job Seekers at these Facilities.

		Male		Female		Total		*p*	F5	*p*	EQMS	*p*
		N = 146	%	N = 89	%	N = 235	%		N = 130		N = 4,500	
Age	< 65 years old	80	54.8%	46	51.7%	126	53.6%	0.643	55.4%	0.746		
Education	< college graduate	90	63.4%	59	67.8%	149	65.1%	0.494	59.5%	0.469	58.8%	<0.001***^c^
Annual Household Income	< 2 million JPY	52	41.9%	25	34.2%	77	39.1%	0.285	34.1%	0.795	23.6%	<0.001***^c^
Life Circumstance	≤ slightly painful	75	51.7%	42	47.7%	117	50.2%	0.554	45.3%	0.394	25.4%	<0.001***^c^
Social Contact	none (isolated)	40	32.0%	5	6.8%	45	22.7%	<0.001***	20.0%	0.844	19.6%	0.283***^c^
Other Social Participation	none	78	54.5%	37	43.5%	115	50.4%	0.108	44.6%	0.429	36.1%	<0.001***^c^
Living With Someone	no (living alone)	38	26.0%	26	29.2%	64	27.2%	0.595	27.1%	0.983	23.4%	0.183***^c^
Self-rated Health	< "cannot say"	22	15.1%	6	6.7%	28	11.9%	0.056	11.5%	0.915	22.7%	<0.001***^c^
WHO5 (mental health)	< 13 points	48	33.1%	16	18.6%	64	27.7%	0.017*	25.6%	0.733		
Reason for Job Seeking	earning living expense	111	76.0%	63	70.8%	174	74.0%	0.374				
	paying debts	14	9.6%	0	0.0%	14	6.0%	0.001**^a^				
	getting allowance	36	24.7%	20	22.5%	56	23.8%	0.703				
	maintaining health	63	43.2%	45	50.6%	108	46.0%	0.269				
	well-being	42	28.8%	45	50.6%	87	37.0%	0.001**				
	social participation	40	27.4%	37	41.6%	77	32.7%	0.025*				
	plenty of time	33	22.6%	25	28.1%	58	24.7%	0.344				
	recomendation by family	8	5.5%	0	0.0%	8	3.4%	0.026*^a^				
Experience of use of other services before ASESC	Hello-Work	103	70.5%	51	57.3%	154	65.5%	0.339	64.6%	0.860		
	SHRC	39	26.7%	13	14.6%	52	22.1%	0.079	19.2%	0.516		
	others	29	19.9%	18	20.2%	47	20.0%	0.956	22.3%	0.603		
Job seeking period before ASESC	months	6.68		4.23		5.75		0.067^b^					

WHO5: World Health Organization 5-item well-being index

* p < .05

**p < .01

***p < .001 all by Pearson's chi-square test, but "a" of Fisher's exact test, “b” of t-test, and “c” of F-test by ANCOVA with 2 covariates of age and sex.

To confirm the attrition bias (survivorship bias), we compared the basic attributes of BL and F5 in Table 1. But we couldn’t find any significant difference. Additionally, to show the sample selection bias of this survey, we compared the baseline data with the data of the exhaustive questionnaire mail survey (EQMS), which was conducted in the neighborhood of one of the targeted ASESCs in July and August of 2015. The respondents were 8,075 older people aged 65 and over who were all living in the district, excluding the elderly living in institutional care facilities and registered as Care-Level 4 whose criteria was partially dependent in basic activities of daily living such as toileting or feeding. In this survey, the number of people with valid responses was 4,500 (55.7%). Comparing the baseline data with that of EQMS by one-way ANCOVA (Analysis of Covariance) with 2 covariates of sex and age, the percentage of the respondents with low education, low annual income, low social contact, and low other social participation was statistically higher (p<0.001). On the contrary, the proportion of respondents with low self-rated health was smaller (p<0.001), which shows that the people who came to ASESC tended to keep their self-estimation about health status higher than general elderly persons.

**Fig 1:**
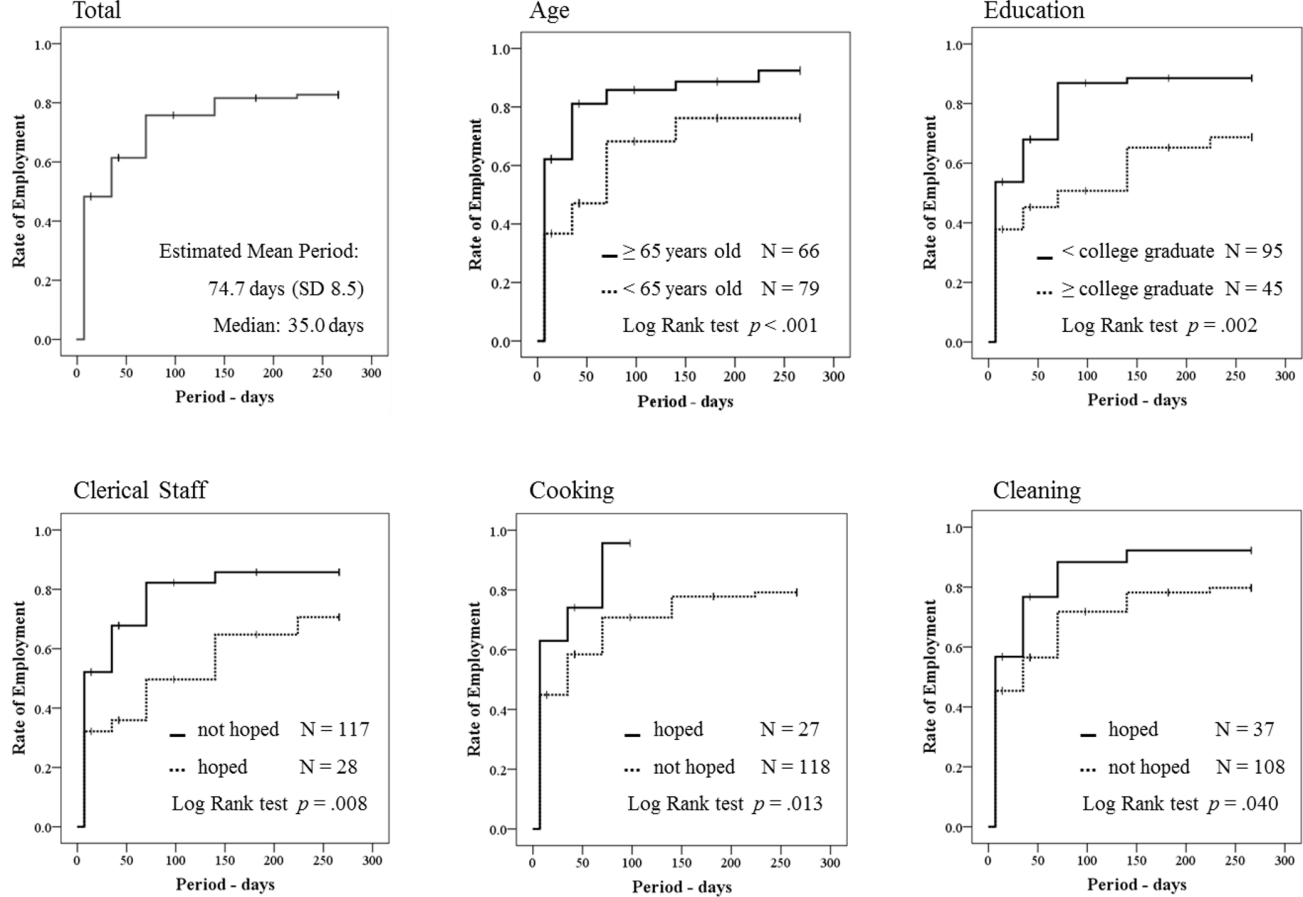
Cumulative Percentage Curve using the Kaplan-Meier Estimator with Log-Rank tests, Rate of Employment vs. Job seeking Period

We analyzed the process of job attainment (rate of employment) by time processing, using the Kaplan-Meier Estimator for each attribute of the 4 basic attributes, 3 life circumstances, 2 health statuses, 8 reasons for job-seeking, and 13 occupations hoped-for beforehand. As a result of Log-Rank tests, we could get statistical significance for the 2 basic attributes of age and education, and the 3 occupations of clerical staff, cooking, and cleaning. Younger elderly job seekers required more days than older ones, as well as those with higher education, or seeking clerical jobs. On the contrary, the persons who hoped to get cooking or cleaning jobs, found it easier to get new jobs. In the targeting span of 38 weeks, 76.2% (131/172) of respondents could get a new job, and the total estimated mean period to do so was 74.7 days (SD 8.5) and the median was 35.0 days (Fig 1).

We confirmed the attributes which affected rate of employment by Cox proportional-hazard regression analysis and obtained 2 significant attributes, age and education (Table 2).

**Table 2:** Results of Cox Proportional-Hazard Regression Analysis.

	Reference Category		B	SE	Wald	*p*-value	Hazard ratio	95% C.I.
Sex	Male	Female	-.037	.228	.026	.872	.964	0.617–1.507
Age	< 65	≥ 65	.566	.214	7.007	.008**	1.761	1.158–2.676
Education	< College Graduate	≥ College Graduate	-.586	.242	5.832	.016*	.557	0.346–0.896
Annual Household Income	< 2 Million JPY	≥ 2 Million JPY	.262	.238	1.217	.270	1.300	0.816–2.070
Social Contact	Isolated	Not Isolated	.237	.260	.833	.361	1.267	0.762–2.109
Social Participation	No	Yes	.035	.212	.027	.868	1.036	0.683–1.570
Living With Someone	No	Yes	-.394	.250	2.472	.116	.675	0.413–1.102
Self-rated Health	< somewhat good	≥ somewhat good	.227	.407	.313	.576	1.255	0.566–2.875
WHO5	< 13	≥ 13	-.055	.246	.051	.822	.946	0.584–1.532

JPY: Japanese Yen, C.I.: Confidence Interval, WHO5: World Health Organization 5-item well-being index

* p < .05

**p < .01.

**Fig 2:**
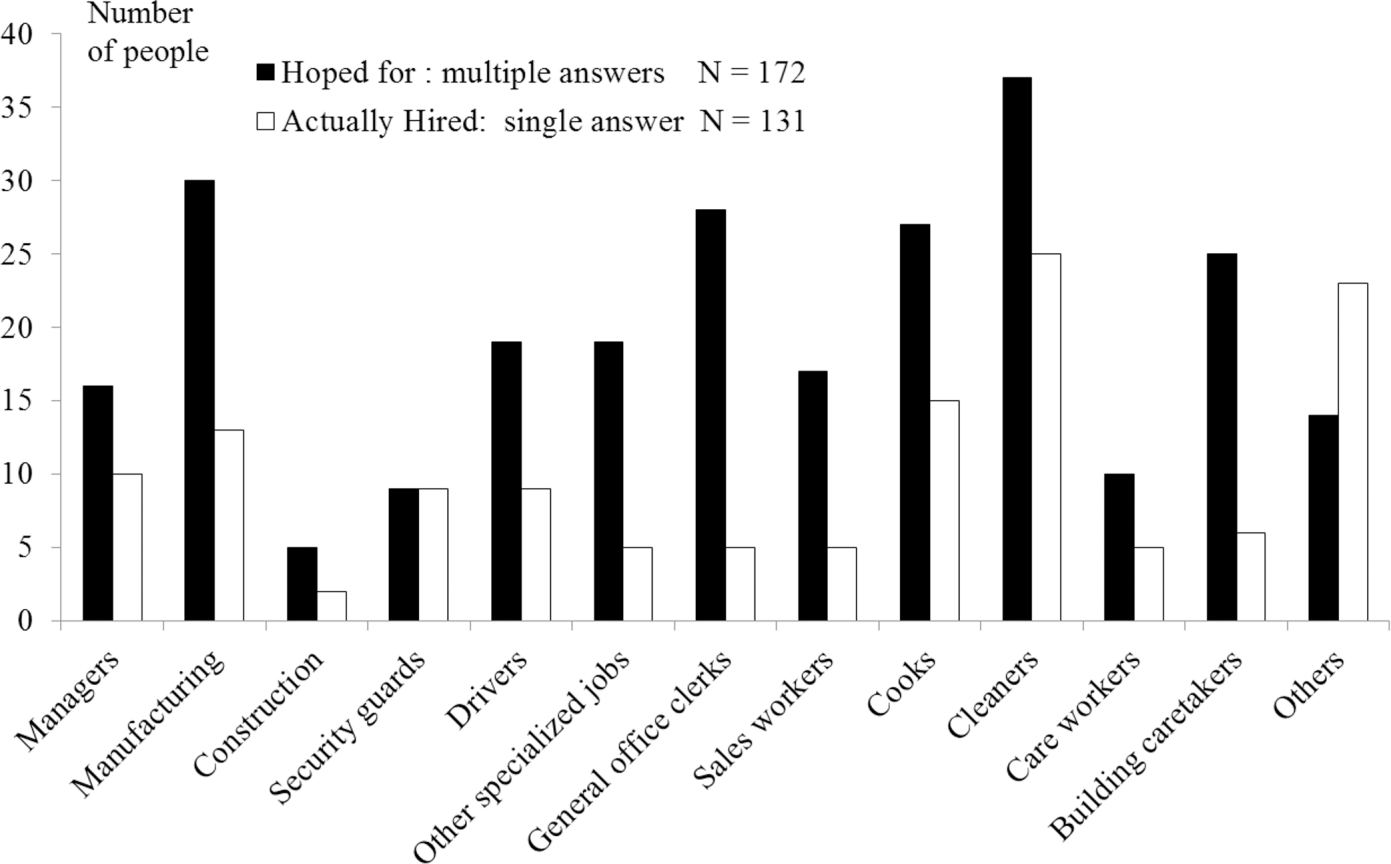
Number of people hoped-for beforehand vs. hired actually, for each occupation

Finally, we compared the numbers of “hoped-for beforehand” and “hired actually” for each occupation (Fig 2). The most popular hoped-for occupation was cleaning, followed by manufacturing, whereas the most hired occupation was cleaning followed by others, cooking, and manufacturing. The percentage of people who could be hired as the same occupation as hoped-for beforehand was 60.3% (79/131).

## Discussion

In this study, the older job seekers at these ASESCs tended to have a relatively low education level, low income, and were seeking jobs for earning living expenses rather than for well-being. Also, it is clear that many of them were in a poor state of mental health. These tendencies are in accordance with our previous report [23]. Half of these job seekers found employment in as short a period as about 35.0 days if they met the conditions and applied to the jobs. An additional 23.8% couldn’t find a job and stayed in that condition for a long time. It was especially difficult for younger and high educational level older job seekers to find jobs. This tendency is completely opposite to the general labor market where these attributes are regarded as advantageous. Typical white-collar workers who were seeking clerical jobs couldn’t find jobs easily.

### Implications

According to these results, we can point out the evaluation and expectations of ASESC.

Firstly, we can say that these local facilities are participating as a complement to classic national employment support systems comprised of Hello-Work and SHRC to assist older job seekers who are mainly seeking jobs for earning living expenses, which can be attained in a short time span and can enable them to earn some money.

Secondly, the older job seekers at ASESCs include people who are isolated, who are in a poor economic situation, and who have poor mental health. There is a previous study showing that more women tend to participate in volunteer activities and that men tend to be easily isolated [24]. Also, there are many previous studies about job loss leading to poor mental health [25]. It is conceivable that they were overly qualified for the jobs they applied for and suffered numerous rejections, they were not able to perform the types of jobs that were readily available, such as cooking or cleaning, or because of social expectations that men have employment and the social prejudice they internalize when they tell people they do not have employment. In these processes, they may suffer a loss of personal identity, harming their dignity. Isolation may be a reason to be nervous, and poor mental health may be a reason for failure to find employment. It may be a vicious circle.

In Japan, the proportion of elderly people is the largest in welfare recipients. They are also regarded as being very similar to the characteristics of the people who are at risk of suicide due to age, economic situation, or lack of supportive acquaintances [26][27][28][29]. From the view point of community-based health and welfare, the preventive interventions by every means are required for such risks [30]. But, it is difficult for the community health workers to expect such unemployed older adults to participate in the regional health services, like a routine medical check-up or other community involvements proactively. Therefore, these facilities are expected to be a consulting service, not only for employment, but also for general living, because it is a very important place for society to keep in contact with these kinds of people. The office clerk at these facilities is also expected to be a gatekeeper for social isolation or suicide for these older people. We have to enlighten these office clerks through appropriate education and training programs about this [31]. It also may be effective to place telephone lifeline posters at the windows, or to organize mutual self-help groups for these older job seekers, especially for those who stay a long time at ASESC.

Thirdly, a mismatch occurs in ASESC, in that the younger group with higher education cannot find appropriate occupations. They may be selective about choosing employment; for example, looking for managerial jobs, specialized jobs, or jobs as clerical staff which are suitable for their higher quality and valuable skills and backgrounds. This selective process takes a long time for them to understand the reality that there are hardly any of these kinds of good job offers. It is more likely that ASESC is the final option for people who couldn’t find a job for a long time. It seems very hard to find a job by other ways as things stand.

As mentioned before, the number of people who have experienced willing or unwilling early retirement is increasing in Japan. The number of people who have to give up their jobs to take care of their family members will also continue increasing with the progress of the super-aging society. Under uncertain environments, many enterprises reduce the number of regular workers and substitute them with non-regular workers. It seems very hard to find room for people, especially older adults who have once left an organization, to be treated well again in the seniority system. So, from the view point of community-based health and welfare, we expect our society to create a social inclusion system to encourage and hire these kinds of people again, not to leave them alone being isolated and aggravating their own health and the community health in neighborhoods. To begin with, the employers can do more to give people who are retrenched or retired more information on employment support services that they can use. They need to do so immediately after the employees leave the companies, so that the time between jobs is not so long and these older persons do not spend a long time experiencing social isolation. It is also important to establish a reeducation system in companies for placement of older human resources [32][33][34], developing suitable and flexible styles of employment [35] or workplaces especially for older workers [36][37], evaluation system for insights into the work of older people [38], or accumulate and generalize tips for how to manage older workers [39].

At the same time, the office clerk at these facilities should encourage these people to reconsider the value of social contributions, instead of only office work, or the value of money. For example, working at social welfare corporations or non-profit organizations is easier to accept for older people than working in a harsh, competitive environment [40]. Fortunately, these places are always short of manpower and there is expected to be a large quantity of job opportunities herein, as the leaders of care prevention, everyday life support synthesises business, due to the 6th plan of the nursing-care insurance system of the Ministry of Health, Labour and Welfare, started from April, 2015 [41]. These types of jobs are rapidly increasing due to recent governmental policies are regarded to be activated at SHRC or ASESC because these are closely related to the community area. Especially SHRC, which is the national network to promote regional activities, looks suitable, but it is currently prohibited to avoid squeezing on private-sector businesses within 20 hours in a week. Therefore, until the removal of the regulation, ASESC will play a leading part to recruit older people in these fields.

In these workplaces, working older people would be highly appreciated by the people involved and they could become familiar with the welfare services they themselves must depend on in the near future. At the same time, it is important to create an atmosphere of respect for these working older people engaging in these fields by the whole of society to sustain their dignity. Services like child-care support or daily life support means indirect support for younger working generations. It would lead to a sustainable, virtuous circle of intergenerational mutual support in the long run, as when the younger generations become older, they, in turn, can support the next younger generation.

### Limitations

There are some limitations in this study. As previously mentioned, this study sample doesn’t represent all older job seekers in Tokyo but the visitors at ASESC. We need further surveys about Hello-Work, SHRC, or other means to consider about the total job seeking process of older adults. Additionally, we need further study to discuss the coordination of these three networks for older job seekers and the optimal ways to combine them, suitable for the situation of each municipality [42]. This study is also limited to the urban area of Tokyo Metropolitan City. In rural areas, agriculture, fishery, and forestry are the main occupations of older workers [43]. The process of job-seeking would be completely different from urban areas. We need further research to investigate the national policy in rural areas.

Furthermore, we need another survey about enterprises, including social welfare corporations and non-profit organizations, which hire older people to give a perspective from the opposite side, to reinforce this study. It is also hoped to prepare objective evidence about the physical [44] or cognitive [45][46] capabilities of older workers, to prove their ability to work, or a measurement system for the suitability or the risk [47][48][49] of engaging in each occupation [50][51]. It is also important to consider the exchange, transfer, or conflict in the workplace between generations [52] for sustainability of the organizations, or of our society.

### Future direction

In the progress of a super-aging society, working in older age is expected not only for economic productivities but also as an activity of social participation for health promotion or preventive care, as a part of the integrated community care system, which the Japanese government is promoting for 2025 [53]. The Japan Geriatric Society also announced that today’s elderly compared with those 10–20 years ago are regarded as being 5–10 years younger in Japan. It will become more and more important to prepare opportunities for these older workers to play active roles in our society, complementarily and beneficially, with health and welfare measures as a whole.
