# Correction: Older Adults Looking for a Job through Employment Support System in Tokyo

**DOI:** 10.1371/journal.pone.0163000

**Published:** 2016-09-09

**Authors:** The PLOS ONE Staff

In Table 1, the final column’s p values for “Social Contact” and “Living With Someone” are incorrect. Please see the corrected Table 1 here. The publisher apologizes for the error.

**Table 1:** Baseline Characteristics of Older Job Seekers at these Facilities.

		Male		Female		Total		*p*	F5	*p*	EQMS	*p*
		N = 146	%	N = 89	%	N = 235	%		N = 130		N = 4,500	
Age	< 65 years old	80	54.8%	46	51.7%	126	53.6%	0.643	55.4%	0.746		
Education	< college graduate	90	63.4%	59	67.8%	149	65.1%	0.494	59.5%	0.469	58.8%	<0.001***^c^
Annual Household Income	< 2 million JPY	52	41.9%	25	34.2%	77	39.1%	0.285	34.1%	0.795	23.6%	<0.001***^c^
Life Circumstance	≤ slightly painful	75	51.7%	42	47.7%	117	50.2%	0.554	45.3%	0.394	25.4%	<0.001***^c^
Social Contact	none (isolated)	40	32.0%	5	6.8%	45	22.7%	<0.001***	20.0%	0.844	19.6%	0.283^c^
Other Social Participation	none	78	54.5%	37	43.5%	115	50.4%	0.108	44.6%	0.429	36.1%	<0.001***^c^
Living With Someone	no (living alone)	38	26.0%	26	29.2%	64	27.2%	0.595	27.1%	0.983	23.4%	0.183^c^
Self-rated Health	< "cannot say"	22	15.1%	6	6.7%	28	11.9%	0.056	11.5%	0.915	22.7%	<0.001***^c^
WHO5 (mental health)	< 13 points	48	33.1%	16	18.6%	64	27.7%	0.017*	25.6%	0.733		
Reason for Job Seeking	earning living expense	111	76.0%	63	70.8%	174	74.0%	0.374				
	paying debts	14	9.6%	0	0.0%	14	6.0%	0.001**^a^				
	getting allowance	36	24.7%	20	22.5%	56	23.8%	0.703				
	maintaining health	63	43.2%	45	50.6%	108	46.0%	0.269				
	well-being	42	28.8%	45	50.6%	87	37.0%	0.001**				
	social participation	40	27.4%	37	41.6%	77	32.7%	0.025*				
	plenty of time	33	22.6%	25	28.1%	58	24.7%	0.344				
	recomendation by family	8	5.5%	0	0.0%	8	3.4%	0.026*^a^				
Experience of use of other services before ASESC	Hello-Work	103	70.5%	51	57.3%	154	65.5%	0.339	64.6%	0.860		
	SHRC	39	26.7%	13	14.6%	52	22.1%	0.079	19.2%	0.516		
	others	29	19.9%	18	20.2%	47	20.0%	0.956	22.3%	0.603		
Job seeking period before ASESC	months	6.68		4.23		5.75		0.067^b^				

WHO5: World Health Organization 5-item well-being index

* p < .05

**p < .01

***p < .001 all by Pearson's chi-square test, but "a" of Fisher's exact test, “b” of t-test, and “c” of F-test by ANCOVA with 2 covariates of age and sex.
